# DNA methylation links genetics, fetal environment, and an unhealthy lifestyle to the development of type 2 diabetes

**DOI:** 10.1186/s13148-017-0399-2

**Published:** 2017-10-03

**Authors:** Emma Nilsson, Charlotte Ling

**Affiliations:** Department of Clinical Sciences, Epigenetics and Diabetes Unit, Lund University Diabetes Centre, Scania University Hospital, Jan Waldenströms gata 35, 205 02 Malmö, Sweden

**Keywords:** Epigenetics, DNA methylation, Type 2 diabetes, Insulin resistance, Aging, Obesity, Intrauterine environment, Genetics

## Abstract

Type 2 diabetes is a complex trait with both environmental and hereditary factors contributing to the overall pathogenesis. One link between genes, environment, and disease is epigenetics influencing gene transcription and, consequently, organ function. Genome-wide studies have shown altered DNA methylation in tissues important for glucose homeostasis including pancreas, liver, skeletal muscle, and adipose tissue from subjects with type 2 diabetes compared with nondiabetic controls. Factors predisposing for type 2 diabetes including an adverse intrauterine environment, increasing age, overweight, physical inactivity, a family history of the disease, and an unhealthy diet have all shown to affect the DNA methylation pattern in target tissues for insulin resistance in humans. Epigenetics including DNA methylation may therefore improve our understanding of the type 2 diabetes pathogenesis, contribute to development of novel treatments, and be a useful tool to identify individuals at risk for developing the disease.

## Background

Type 2 diabetes is one of the most common chronic metabolic diseases in developed countries [1]. This form of diabetes is a consequence of the target tissues becoming resistant to the effects of insulin and the failure of pancreatic β-cells to produce enough insulin. It is shown that type 2 diabetes develops with age, physical inactivity, and obesity in subjects with a genetic predisposition and/or in subjects who have experienced an adverse intrauterine environment. It is a complex multifactorial disease whose development is dependent on interactions in and between the predisposing factors.

One link between genes, environmental exposure, and disease development is epigenetics. It provides a molecular mechanism to explain how interactions between genetic and environmental factors may be involved in a disease process. The term epigenetics is typically described as heritable changes in gene function that occur without a change in the nucleotide sequence [2]. Epigenetic regulation includes DNA methylation, histone modifications, and non-coding RNA. DNA methylation is the most studied epigenetic mark so far and occurs mainly at the fifth position of the cytosine ring in CpG dinucleotides. DNA methylation is required to maintain cell-specific gene expression, plays an important role during embryonic development, and contributes to the establishment of imprinting and X-chromosome inactivation [3–5]. DNA methylation in promoter regions has been associated with transcriptional silencing. However, emerging data show that the effect of DNA methylation depends on the genomic location, and it may also affect alternative splicing, genomic stability, transcriptional elongation, and transcription of non-coding RNAs [3]. It may thereby also be associated with increased gene expression. The establishment and maintenance of epigenetic modifications are susceptible to environmental factors including dietary factors and changes in metabolism [6]. This, in addition to the fact that epigenetic changes accumulate in the living individual [7], led to the hypothesis that epigenetic modifications could be involved in age and lifestyle-related metabolic diseases such as type 2 diabetes. Indeed, we and others have shown altered epigenetic states in tissues important for glucose homeostasis including pancreas, liver, skeletal muscle, and adipose tissue from subjects with type 2 diabetes compared with nondiabetic controls [8–14]. These studies include differential DNA methylation of candidate genes that affect insulin secretion from pancreatic beta cells and thereby could play an important role in the pathogenesis of type 2 diabetes [8, 14].

**Fig. 1:**
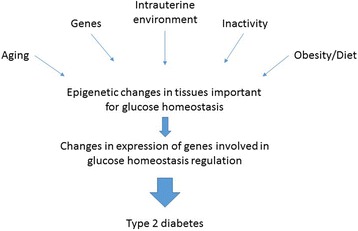
A suggested model of type 2 diabetes development and focus of this review article. Risk factors for type 2 diabetes affect expression of genes involved in glucose homeostasis regulation via epigenetic modifications including DNA methylation

The aim of this review is to summarize studies supporting epigenetics as a link between genetics, the fetal environment, an unhealthy lifestyle and the development of type 2 diabetes. In particular, this review will focus on risk factors for type 2 diabetes and their impact on DNA methylation in target tissues for insulin resistance in humans, including skeletal muscle, liver and adipose tissue (Fig. 1).

## DNA methylation and risk factors for type 2 diabetes

### Intrauterine environment

Fetal programming describes the process by which different environmental conditions during development can have long lasting effects on metabolic pathways influencing disease susceptibility. The first evidence for the importance of the intrauterine environment in relation to type 2 diabetes came in the early 1990s [15, 16]. Since then, numerous studies have reported a relationship between low or high birth weight and risk of developing type 2 diabetes later in life [17–20]. Already young adult individuals born with low birth weight show signs of metabolic impairment related to insulin resistance including an altered fat distribution, increased lipolysis, and reduced expression of key insulin signaling proteins in insulin target tissues [21–24]. The increased risk of insulin resistance and type 2 diabetes in these individuals has long been speculated to originate at the epigenetic level, since epigenetic marks are thought to respond to the surrounding environment. Prenatal factors including mode of conception, maternal smoking, and maternal diet have indeed shown to effect the DNA methylation pattern [25–27]. Differential methylation of *IGF2*, as well as other type 2 diabetes-related genes, have been found in children born to mothers experiencing famine, further pointing to epigenetics as a mechanism connecting prenatal nutrition and the onset of type 2 diabetes later in life [28–30]. Also, periconceptual folic acid supplementation has been shown to alter the methylation in *IGF2* in children from the folic acid-supplemented mothers [31]. Godfrey et al. reported that epigenetic marks measured in umbilical cord tissue at birth can predict phenotypic outcomes such as obesity in later childhood [32]. We have shown that young men who had a low birth weight have differential DNA methylation in their adipose tissue compared with men born with a normal birth weight [33]. A recent study also suggests that altered epigenetic and transcriptional regulation of adipose-derived stem cells could play a role in programming adipose tissue dysfunction in individuals born with low birth weight [34].

The phenotypic effects of epigenetic modifications during development may not manifest until later in life in response to environmental challenges such as energy dense diets. As an example of this, we found that 5 days high-fat overfeeding unmasked a decreased plasticity in subjects born with low birth weight compared with subjects born with normal birth weight at the DNA methylation level in the skeletal muscle [35, 36]. Also, fasting induces DNA methylation changes in *LEP* and *ADIPOQ* promoters in adipose tissue among normal birth weight but not among low birth weight subjects. The altered epigenetic flexibility in low birth weight subjects might contribute to their differential response to fasting and increased risk of metabolic disease [37].

### Genes

The genetic contribution to type 2 diabetes has been demonstrated with linkage analyses and twin and adoption studies [38]. However, our knowledge about the impact of environmental factors is so far greater than the understanding of the underlying genetic factors [39]. Association studies have until now identified > 120 genomic loci influencing type 2 diabetes susceptibility [40]. A large number of the identified single nucleotide polymorphisms (SNPs) are located in intergenic regions, in introns, and/or are not predicted to result in functional changes at the protein level. The molecular mechanisms through which these SNPs influence gene function and disease pathology are largely unknown but may include epigenetic mechanisms. The introduction or removal of a CpG site may be a molecular mechanism through which some of the type 2 diabetes SNPs affect gene function. Indeed, a study investigated the 40 SNPs associated with type 2 diabetes at that time and found that 19 of 40 (48%) SNPs introduce or remove a CpG site [41]. In adipose tissue, the degree of methylation seems to mediate the impact of SNPs on metabolic traits, including insulin sensitivity [42]. In the skeletal muscle, we further showed that interactions between genetic (SNP), epigenetic (DNA methylation), and non-genetic (age) factors influence gene expression and metabolism [43].

We have observed differential DNA methylation in the skeletal muscle from individuals with a family history of type 2 diabetes compared with individuals without such family history [11]. Although the epigenetic differences we find between these individuals may be due to genetic factors, they could of course also be due to a shared environment within families. However, based on heritability estimates in twin and family studies, several studies have found that DNA methylation levels are under genetic control [12, 44–47]. We also found support for a genetic impact on DNA methylation in a study where we found a stronger correlation of genome-wide adipose tissue DNA methylation levels in monozygotic twin pairs compared with same-sex dizygotic twins or unrelated subjects [9].

### Diet

Methyl groups for all biological methylation reactions are primarily supplied from dietary methyl donors and co-factors such as folate, choline, methionine, vitamin B_2_, vitamin B_6_, and vitamin B_12_. These dietary components can affect the one-carbon metabolism that determines the amount of *S*-adenosylmethionine (SAM), which is the methyl donor for DNA and histone proteins. The reliance on dietary sources of methyl donors for DNA methylation reactions has led to the suggestion that nutrients may affect both the establishment and maintenance of DNA methylation patterns. Subjects with type 2 diabetes seem to have reduced folate levels in serum compared with healthy controls [10]. Interestingly, at the same time, the subjects with type 2 diabetes also had reduced DNA methylation of the majority (94%) of differentially methylated CpG sites in a genome-wide analysis in the human liver [10]. Folate levels correlated negatively with fasting glucose levels already in nondiabetic subjects, suggesting that reduced circulating folate levels may contribute to the development of type 2 diabetes.

Energy-dense food high in calories and fat is an important risk factor for obesity and type 2 diabetes [48]. A study investigating the effect of a short-term (5 days) high-fat overfeeding diet on the genome-wide DNA methylation pattern in the skeletal muscle from healthy young men found widespread DNA methylation changes affecting almost half of the investigated genes [49]. Due to the cross-over design of the overfeeding study, the reversibility of the induced DNA methylations could be investigated. The overfeeding-induced methylation changes were only partly reversed 6–8 weeks after returning to the control diet. The slow reversibility could have implications for build-up of CpG methylation over time. In a recent randomized control study, we investigated the impact of 7 weeks of saturated or polyunsaturated fat overfeeding on the DNA methylation pattern in human adipose tissue and observed distinct epigenetic changes induced by the two different overfeeding diets [50]. Both diets resulted in a similar weight gain and an increased mean degree of methylation in adipose tissue. However, the saturated fat diet resulted in elevated liver and visceral fat accumulations. Also, these data suggest that DNA methylation at baseline can predict weight gain in response to overfeeding. In addition, DNA methylation in adipose tissue seems to be able to predict weight loss in response to a low caloric diet as well [51, 52].

### Obesity

Increased body fat stores resulting from an imbalance between energy intake and energy expenditure characterize obesity. The prevalence of overweight and obese individuals has increased dramatically in the last decades. Obesity constitutes a major risk factor for several serious diseases including type 2 diabetes. Studies focusing on epigenetic patterns in obesity have found altered DNA methylation levels in genes related to metabolic processes, for example, circadian clock system genes [53]. Because obese people are at increased risk of many age-related diseases, it is a possible hypothesis that obesity increases the biological age of some tissues. Horvath et al. reported a strong correlation between high body mass index (BMI) and the epigenetic age of liver tissue, suggesting that the accelerated epigenetic aging may play a role in liver-related comorbidities of obesity, such as insulin resistance [54]. BMI was related to DNA methylation in whole blood cells from 479 individuals and identified CpG sites annotated to *HIF3A* with increased DNA methylation associated with increased BMI. The association was further validated in adipose tissue from 635 females [55]. It has been proposed that the HIF system could play a role in mechanisms involved in adipose tissue inflammation, insulin resistance, and the etiology of obesity-related diseases [56, 57]. We analyzed the genome-wide DNA methylation pattern in human adipose tissue from 96 males and 94 females and found that DNA methylation of ~ 5000 CpG sites was associated to BMI [58]. The strong effect of increased BMI on the degree of DNA methylation in human adipose tissue indeed propose that obesity can mediate some of its effects via altering the epigenome. Interestingly, a large number of these CpG sites do also show differential DNA methylation in adipose tissue from subjects with type 2 diabetes compared with controls [9], suggesting that BMI-associated changes in DNA methylation may predispose to type 2 diabetes. We could also link BMI-associated DNA methylation to differential expression of almost 3000 genes [58]. Another epigenome-wide study showed that BMI is associated with widespread changes in DNA methylation in blood and that these changes correlated to methylation patterns in other tissues including subcutaneous and omental fat, liver, and muscle. They demonstrated that the DNA methylation alterations are a consequence rather than a cause of obesity. The methylation changes occurred in genes involved in lipid metabolism and inflammation and predicted future development of type 2 diabetes [59]. DNA methylation and gene expression patterns in adipose tissue have also been shown to differ significantly within young adult monozygotic BMI-discordant twin pairs [60]. We have in a recent study identified abnormal epigenetic changes during differentiation of skeletal muscle stem cells from obese compared with non-obese humans, proposing an altered epigenetic memory in muscle stem cells due to obesity [61]. Interestingly, there were many genes involved in epigenetic regulation and metabolic diseases among genes showing differential methylation and expression during differentiation in obese subjects only.

### Inactivity

Physical inactivity contributes to lifestyle-related diseases, including obesity and type 2 diabetes. We have previously shown that 9 days of bed rest induce DNA methylation changes in *PGC1α* that are not totally reversed after a retraining period of 4 weeks in the skeletal muscle of healthy young men [62]. PGC1α is the master regulator of mitochondrial biogenesis, and *PGC1α* mRNA expression is reduced in the skeletal muscle from subjects with type 2 diabetes [63]. Interestingly, it has been shown that DNA methylation regulation of *PGC1α* in the skeletal muscle may explain interindividual variation in response to exercise training [64]. Although the benefits of regular exercise are well known, the underlying regulatory mechanisms are still not completely understood but may include epigenetics. Methylation changes in insulin target tissues have been reported after both acute and long-term physical exercise [11, 65–68]. A few years ago, it was shown that DNA methylation might be involved in muscle adaptation to regular exercise training in men with or without a family history of type 2 diabetes [11]. A 6-month exercise intervention changed both DNA methylation and mRNA expression of a number of genes involved in skeletal muscle metabolism. A study of fat biopsies from the same individuals showed that exercise induces genome-wide changes in DNA methylation also in human adipose tissue [66]. Here, a general global increase in DNA methylation in addition to changes in DNA methylation of 17,975 individual CpG sites including several genes associated with type 2 diabetes and obesity was observed. Two genes with differential methylation and expression in response to exercise, *Hdac4* and *Ncor2*, were silenced in 3T3-L1 adipocytes which resulted in increased lipogenesis both in the basal and insulin-stimulated state. The epigenetic changes induced by bed rest or exercise may hence affect tissue function and predispose to or protect against disease risk (Table 1).

**Table 1:** Summary of some studies that have investigated the impact of physical inactivity and/or activity on DNA methylation in human skeletal muscle and adipose tissue

Intervention	Tissue	Key findings	Reference
9 days of bed rest	Muscle	Increased methylation of *PGC1α*	[62]. AC Alibegovic et al., American journal of physiology Endocrinology and metabolism 2010;299:E752-763
Acute exercise	Muscle	Response to exercise based on changed methylation of *PGC1α*	[64]. S Bajpeyi et al., Endocrinology 2017;158:2190-2199
6 months exercise intervention	Muscle	2051 genes (i.e. *MEF2A*, *RUNX1*, *NDUFC2*, and *THADA*) with decreased and 766 genes with increased methylation	[11]. MD Nitert et al., Diabetes 2012;61:3322-3332
Acute exercise	Muscle	Decreased methylation of *PGC1α*, *PDK4*, and *PPARδ*	[65]. R Barres et al., Cell metabolism 2012;15:405-411
3 months supervised exercise	Muscle	Methylation changes at 4919 sites across the genome in trained leg	[67]. ME Lindholm et al., Epigenetics 2014;9:1557-1569
6 months exercise intervention	Adipose tissue	17,975 individual CpG sites in 7663 unique genes (i.e. *HDAC4* and *NCOR2*) showed altered methylation	[66]. T Ronn et al., PLoS genetics 2013;9:e1003572
16 weeks of either endurance or resistance training	Muscle	Endurance and resistant training induced different epigenetic changes	[68]. DS Rowlands et al., Physiol Genomics 2014;46:747-765

### Aging

**Fig. 2:**
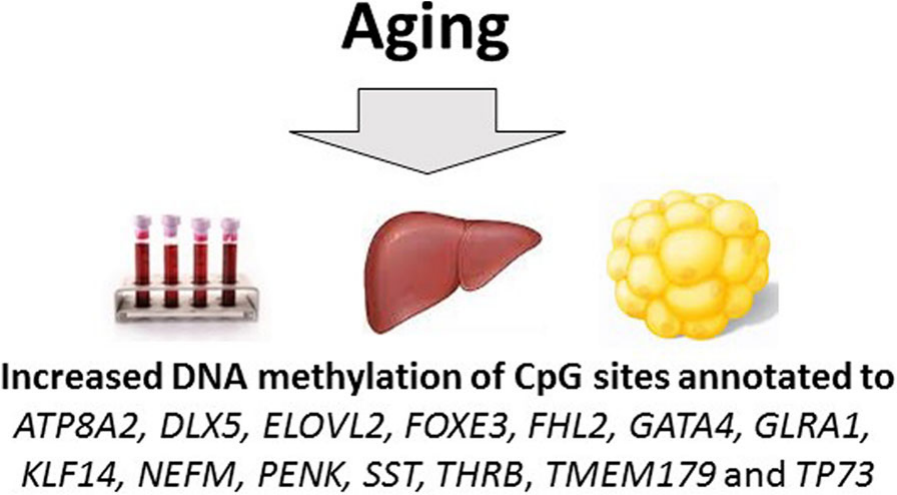
A summary of some tissues and genes that are affected by age by altered DNA methylation

Aging is a complex multifactorial process affecting all living beings. It is associated with a progressive decline in physiological functions and an increased incidence of chronic metabolic diseases including type 2 diabetes. One possible explanation may be that aging causes epigenetic changes that effect expression of genes important for glucose homeostasis. A study of young and elderly monozygotic twin pairs showed that the young twin pairs exhibit a similar epigenetic pattern, whereas intrapair differences were substantial in elderly twin pairs, supporting the idea that epigenetic changes accumulate during life [7]. Studying the skeletal muscle from young and elderly twins, we could show age-related DNA methylation and expression changes of genes encoding proteins involved in the respiratory chain, i.e., *NDUFB6* and *COX7A1* [43, 69]. A prospective study has further shown that DNA methylation changes over time [45]. Epigenetic drift ﻿is suggested to be caused by environmental factors or spontaneous stochastic errors in the process of transmission of DNA methylation and leads to unpredictable differences in the methylome among aging individuals. The imperfect maintenance of epigenetic marks during the process of replication may be accentuated during aging as levels of DNA methyltransferase 1 (DNMT1), the maintenance DNMT, has been reported to decline in aging cells [70]. Several studies have investigated DNA methylation changes with age in target tissues for insulin resistance [58, 71–75]. Although many age-related changes depend on cell type [72], several studies from our group and others have shown that some DNA methylation changes occur at specific sites during aging in a highly reproducible way, independently of tissue type, sex, or disease state [58, 71, 76–80]. A study analyzing ~ 8000 samples representing 51 healthy human tissues and cell types identified that DNA methylation at 353 CpGs accurately predicted age [73]. Another study investigated the rate of DNA methylation change by building a predictive model of the aging methylome from the blood of individuals aged 19–101 years [81]. This study identified a set of 71 CpGs that have a high accuracy of age prediction and nearly all markers occur within or near genes associated with aging. Interestingly, a number of the blood-based epigenetic markers of age can also be found in tissues relevant for glucose homeostasis [58, 71, 76]. These include differential methylation of *ELOVL2*, *KLF14*, and *FHL2* (Fig. 2). Although these studies clearly demonstrate that age affects the DNA methylation pattern, future studies need to address the mechanisms behind these specific age-related epigenetic changes taking place in multiple tissues.

## Conclusions

The prevalence of type 2 diabetes is rapidly increasing worldwide. This disorder causes suffering, deaths, and costs and is becoming a more and more severe problem for our society. It is important to better understand the underlying mechanisms in order to improve prediction, prevention, and treatment of the disease. As suggested in this review, type 2 diabetes develops due to an interplay between epigenetics, genetics, fetal environment, and lifestyle. Studies presented in this review show that DNA methylation is highly dynamic and responsive to the environment. Despite the facts that most of the findings in the reviewed papers are correlative and that most studies investigate only a small number of the existing CpG sites, these findings will be valuable for hypothesis development of future studies. Future work should focus on finding the optimal lifestyle (for example, type of diet and type/duration of exercise) for hindering the development of epigenetic-based diseases and how all these could be affected by genetic background. Epigenetics can either provide a biological mechanism for disease development, be targeted for therapy, or serve as a biomarker of disease or disease risk even if not directly involved in causing the disease. The epigenetic studies summarized in this review may improve our understanding of disease pathogenesis, contribute to development of novel therapies, and improve prediction of type 2 diabetes.

## Abbreviations

BMI: Body mass index
DNMT1: DNA methyltransferase 1
SAM: *S*-adenosylmethionine
SNPs: Single nucleotide polymorphisms
